# MiBalert, a realtime alert of multidrug-resistant bacteria in the electronic medical record

**DOI:** 10.1186/2047-2994-4-S1-P201

**Published:** 2015-06-16

**Authors:** B Olesen, K Rasmussen, M Voldstedlund

**Affiliations:** 1 Task Force for reduction of nosocomial infections, The Capital Region of Copenhagen, Denmark; 2 CIMT, Capital Region of Denmark; 3 Statens Serum Institut, Denmark

## Introduction

When patients are readmitted or transferred between departments or hospitals, information on multiresistant microorganisms is often recognized late or lost, resulting in infection or colonization of fellow patients or even hospital outbreaks. Currently, the hospitals of the Capital region, covering 1.8 million inhabitants, face an outbreak of vancomycin resistant *Enterococcus fæcium* (VRE). All hospitals share the same EHR system.

## Objectives

To establish a nationwide automatic real time alert in electronic health records (EHR) of cases with multiresistant microorganisms. In 2015 a prototype alert was launched alerting cases with VRE in the Capital region. Here we share our first experiences.

## Methods

MiBalert uses data from the Danish microbiological database MiBa ([1]). MiBa receives electronic copies of all microbiological test reports in Denmark. All reports include a unique patient identifier. Each time a patient´s EHR is accessed an automatic request is sent to MiBa automatically activating MiBalert. An electronic alert is generated if a positive test report for VRE has been found within 1 year. MiBalert appears in the EHR as a colored smart button giving 3 different signals:

1. Positive test report of VRE (red MiBAlert symbol)

2. No positivetest reports of VRE (green MiBa symbol)

3. Malfunction (grey X symbol)

By Mouse-over, a text shows the date of the positive VRE identification. A click on the smart button connects the doctor to MiBa, providing directly access to all the patients’ microbiological reports.

## Results

- Staff at the emergency departments found that MiBAlert effectively highlighted VRE cases

- The hygiene team reported of earlier recognition of VRE cases at sector transmissions

- Staff at departments of radiology, medicine and outpatient clinics found MiBAlert very helpful

- The prototype alerting VRE-cases was a success

## Conclusion

- MiBAlert improves patient safety, in particular during sector transmissions

- MiBAlert is a valuable tool fighting the nosocomial VRE outbreak

- MiBAlert is based on a national database and can easily be implemented nationwide

- Soon other multiresistant bacteria will be included in MiBAlert

## Disclosure of interest

None declared.
